# Managing uncertainty in forecasting health workforce demand using the Robust Workforce Planning Framework: the example of midwives in Belgium

**DOI:** 10.1186/s12960-023-00861-1

**Published:** 2023-09-18

**Authors:** Nadia Benahmed, Mélanie Lefèvre, Sabine Stordeur

**Affiliations:** Belgian Health Care Knowledge Centre, Boulevard du Jardin Botanique, 55, 1000 Brussels, Belgium

**Keywords:** Midwifery, Human resources for health, Midwifery services, Scenario planning, Belgium

## Abstract

**Background:**

In Belgium, the Planning Commission for Medical Supply is responsible for monitoring human resources for health (HRH) and ultimately proposing workforce quotas. It is supported by the Planning Unit for the Supply of the Health Professions. This Unit quantifies and forecasts the workforce in the healthcare professions on the basis of a stock and flow model, based on trends observed in the past. In 2019, the Planning Unit asked the KCE (Belgian Health Care Knowledge Centre) to develop additional forecasting scenarios for the midwifery workforce, to complement the standard historical trend approach. The aim of this paper is to present the development of such forecasting scenarios.

**Methods:**

The Robust Workforce Planning Framework, developed by the Centre for Workforce Intelligence in the UK was used to develop alternative midwifery workforce scenarios. The framework consists of four steps (Horizon scanning, Scenario generation, Workforce modelling, and Policy analysis), the first two of which were undertaken by KCE, using two online surveys and five workshops with stakeholders.

**Results:**

Three alternative scenarios are proposed. The first scenario (close to the current situation) envisages pregnancy and maternity care centred on gynaecologists working either in a hospital or in private practice. The second scenario describes an organisation of midwife-led care in hospitals. In the third scenario, care is primarily organised by primary care practitioners (midwives and general practitioners) in outpatient settings.

**Conclusions:**

The Robust Workforce Planning Framework provides an opportunity to adjust the modelling of the health workforce and inform decision-makers about the impact of their future decisions on the health workforce.

**Supplementary Information:**

The online version contains supplementary material available at 10.1186/s12960-023-00861-1.

## Background

### Midwifery in Belgium

In Belgium, the training and practice of midwives are regulated by the Law of 10 May 2015 on the practice of health professions. Although the minimum content requirements for midwifery training are described in this law, each linguistic community may define the duration of the training needed to acquire the required skills, i.e. 3 years in the Flemish Community and 4 years in the French Community.

The law distinguishes between activities performed by midwives with complete autonomy and those that require medical supervision. Autonomous midwifery activities are listed in the law and include pregnancy diagnosis, follow-up of low-risk pregnancies (maternal and child risk assessment, birth preparation, and parent education), eutocic deliveries (including amniotomy, episiotomy, perineal suturing), postnatal care, care of healthy newborns, preventive measures, and emergency procedures. They also have the right to prescribe a limited number of drugs listed in the law. The management of fertility problems, high-risk pregnancies, high-risk deliveries, and newborns with life-threatening conditions requires medical supervision. In addition, the law describes procedures that are explicitly prohibited for midwives, namely: artificial dilation of the cervix; use of forceps and vacuum; administration of anaesthesia (except local anaesthesia for performing or suturing episiotomy); and inducing abortion. Except in emergencies, midwives are also prohibited from performing the following procedures: internal version, breech extraction, manual removal of the placenta and manual exploration of the uterus.

In Belgium, patients are free to choose their care provider and the setting of care. Antenatal care can be provided in a variety of settings, including hospitals, private practices and other centres. The majority of women give birth in hospital while the number of outpatient deliveries (at home, in a birth centre, or in a one-day hospitalisation) has remained fairly stable over time, accounting for about one per cent of the total number of deliveries [1–3]. Early postnatal care is usually provided in hospital, while postnatal care can be provided at home or in other settings (hospital, private practice, etc.).

In 2019, 12 088 midwives were licensed to practise [4], of whom 57% worked in the healthcare sector (7 175 FTEs) [5]. With more than 9 out of 10 births taking place in hospital [1–3], the majority of midwifery activity took place in hospitals. As a result, less than 10% of the midwives worked in an outpatient setting [5].

### Planning for healthcare professionals in Belgium

In Belgium, the planning of healthcare professionals is a responsibility shared by the federal state and the federated entities [6].

The Planning Commission for Medical Supply, under the authority of the Federal Minister of Health, is responsible for monitoring human resources for health (HRH) and proposing the regulation of the workforce through a system of federal quotas. Recently, besides this commission, two commissions have been set up in the federated entities to monitor HRH in their territories. The authorities of the federated entities regulate the candidates for training in order to meet the federal quotas.

The Planning Commission for Medical Supply is supported by the Planning Unit for the Supply of the Healthcare Professions. This Unit quantifies and forecasts the supply of health professionals, including midwives, on the basis of a stock and flow model [7]. Stock refers to the available supply of midwives expressed in terms of headcount and Full Time equivalents (FTE) working in the health sector. Flows are made up of inflows (new entrants to the labour market through graduations, immigration, or return to the profession) and outflows (resignations, dismissals, retirements, or deaths). The forecasting model estimates the future evolution of the workforce using historic trends. Based on demographic trends, the output of the model is the midwife-to-population ratio weighted by the consumption of care [8]. The weighting is done by multiplying the “gross” population projections by a consumption rate, based on the reimbursement of midwifery fees.

In 2019, the Planning Unit charged the KCE (Belgian Health Care Knowledge Centre) to develop additional forecasting scenarios to complement the standard historic trend approach. The mission consisted of developing different scenarios for the management of pregnancy and childbirth to estimate their impact on the future demand for midwives, over a 25-year period.

This paper illustrates both the development and the quantification of alternative forecasting scenarios for the demand for midwives in Belgium.

## Methods

### The Robust Workforce Planning Framework

This Framework was created by the Centre for Workforce Intelligence, which was active between 2010 and 2016 to support the planning for future health workforce needs in the UK [9].

The purpose of the Framework is to focus on the uncertainty that arises in the context of long-term forecasting of healthcare supply and demand. To capture the complexity of factors influencing the workforce supply, the organisation of health care, and the demand for health services, the Robust Workforce Planning Framework consists of a four-step approach [9]:

1. Horizon scanning
  Horizon scanning aims to inform planners of weak or early signals and uncertain factors that would affect both workforce and service demand.
2. Scenario generation
  The output of the horizon scanning process is structured to generate a set of plausible, challenging, and coherent scenarios. The scenario generation process allows for a move from a set of attending issues to a holistic understanding of the future. In other words, the uncertainties identified in the horizon scanning are structured into a set of interrelated variables (scenario script) and their corresponding values (elicitation).
3. Workforce modelling
  The workforce supply is projected and its balance with respect to demand is analysed across the different scenarios generated in the previous stage.
4. Policy analysis
  This final step involves identifying the workforce planning decisions required for each scenario.

The Framework requires a high level of stakeholder involvement and therefore uses participatory methods such as surveys and workshops. It can be complemented by targeted expert review and text mining [10].

### Application of the Robust Workforce Planning Framework for the simulation of demand for the Belgian midwifery

**Fig. 1:**
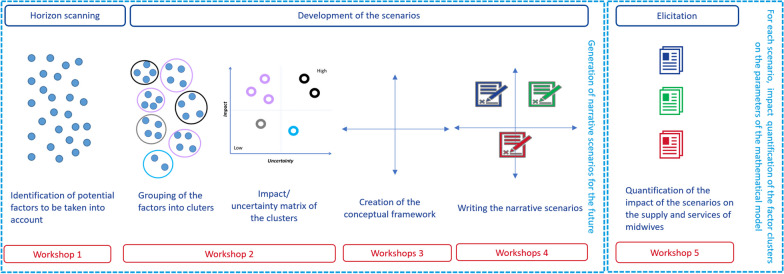
Horizon scanning and scenario generation: methods and outputs

The first two steps of the Framework were carried out in five workshops with 31 invited participants (see Fig. 1). Workforce modelling and policy analysis were then carried out by the Planning Unit. These steps are further summarised in the discussion section.

### Horizon scanning

The horizon scanning was carried out in two phases. First, a preparatory online survey of stakeholders was conducted in French and Dutch. Stakeholders from midwifery professional associations, midwifery schools, the hospital sector, public authorities and administrations, patient associations, and individual professionals (midwives and gynaecologists) were interviewed to identify significant factors to be added to the original forecasting model. The variables included in the original forecasting model, and the description of the participants can be found in Additional file 1. The online survey (see Additional file 1) was sent to 122 participants, with a response rate of 75%. Participants were invited via a call in both French and Dutch. Participants could choose their language before starting the survey. The survey presented 50 factors derived from a preparatory exercise [11] and grouped into 14 domains [12] (see survey in Additional file 1). For each domain, participants were asked to identify factors to add to the initial list.

Results of the survey were then discussed in an initial workshop to assess the relevance and the potential impact of the proposed factors on the future workforce (see Fig. 1 and Additional file 1: Material Sect. S2.3).

### Scenario generation

All workshops were led by an external team (ShiftN) with no prior connection to the participants.

#### Generation of narrative scenarios for the future midwifery practice

The generation of narrative scenarios was performed in a 4-step approach during 3 workshops (see Fig. 1):

1. Factors identified in the horizon scanning exercise were clustered into broader thematic groups (e.g. budget available for health care, tariffs for health services, quality of working conditions of midwives…). The clusters were created by the participants after refining the wording of each factor by consensus and regrouping similar ideas. Consensus was reached through discussion between participants, facilitated by ShiftN.
2. Workshop 2 participants of were asked to place each cluster on an impact/uncertainty matrix. The level of uncertainty for each cluster and the relative strength of each cluster’s potential impact on the workforce had been determined by consensus among the participants in Workshop 2.
3. Focusing on clusters with a high impact on the future midwifery workforce, a conceptual framework for writing narrative scenarios was created. From these clusters, critical uncertainties (i.e. clusters that combine a high impact on the future workforce and a high uncertainty in their realisation; black circles in Fig. 1) and driving forces (i.e. clusters that combine a high impact on the future workforce and a high certainty in their realisation; purple circles in Fig. 1) are of particular interest for generating scenarios for the future. The search for the underlying concepts common to high-impact clusters allows the research team to create/generate a conceptual framework represented by a 4-quadrant matrix.
4. Based on the output of the third workshop, detailed narrative scenarios were developed in small groups (mixed midwives, gynaecologists and stakeholders involved in the organisation of care), based on the conceptual framework. A common template with key elements was used, including a description of the future professional activities of the main actors involved in the management of low-risk and high-risk pregnant women, the creation of care pathways and referral procedures, and the location of midwifery care (hospital, home or birth centre). Finally, scenarios were discussed and refined. Participants were then asked to rate the impact of each scenario element on the future supply and demand for midwifery care (low, medium, or high).

#### Quantification of all parameters included in the scenarios

**Fig. 2:**
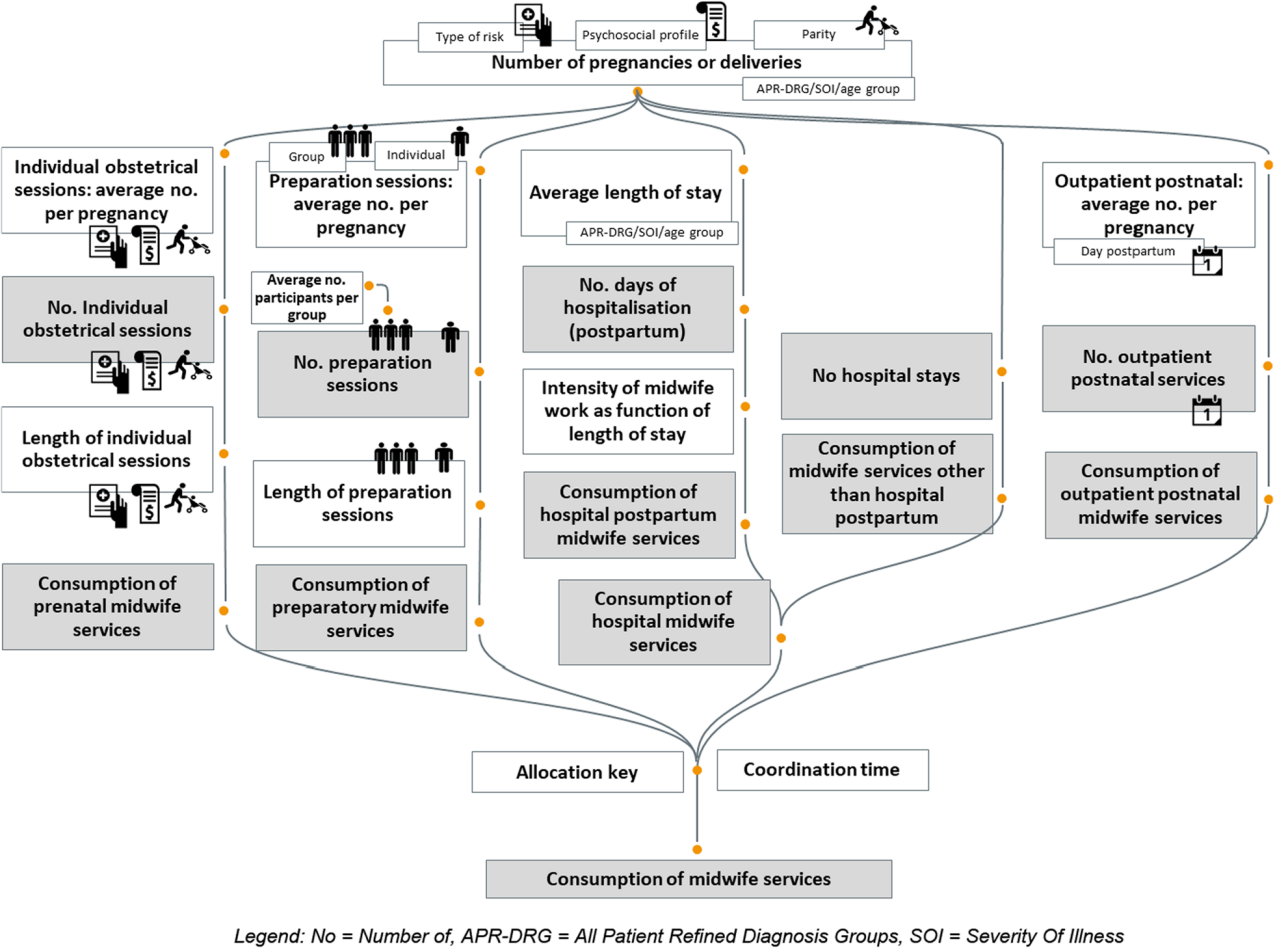
Modelling of the parameters taken into account in the scenarios to quantify (the evolution of) care consumption. No, number of; APR-DRG, all patient refined diagnosis groups; SOI, severity of illness

Based on the narrative of each scenario, the evolution of the consumption of midwifery care was evaluated using the model illustrated in Fig. 2. Midwifery services were categorised into 5 pillars, i.e. antenatal obstetric sessions, birth preparation sessions, hospital care (delivery and postnatal care), and outpatient postnatal care. These pillars were derived from the description of midwives’ activities in the narrative scenarios and from existing categorisations in various administrative databases in Belgium.

Other activities not reported in Fig. 2 (related to fertility, neonatology, research, etc.) were assumed to remain stable over time and therefore not influenced by the organisational models described in the different scenarios.

Each rectangle in Fig. 2 represents a parameter of the model. Where relevant, differentiated parameters are defined according to level of risk during pregnancy, parity, mode of delivery, etc. For example, the average number of obstetric sessions is expected to be different according to risk level, the psychosocial profile, and the parity. Similarly, the average length of hospital stay is different for caesarean sections than for vaginal births, varies according to the age of the patient, etc.

A numerical value was assigned to the evolution of each parameter included in the model. Wherever possible, assumptions were substantiated by objective data and projections. In order to assign a numerical value to a parameter for which no quantitative data were available, an elicitation process was used, requiring the judgments of experts in the field. An elicitation group was therefore set up, consisting of experts from different backgrounds (midwives, gynaecologists, medical and social workers, hospital representatives, and representatives of midwifery schools). Of the 74 experts contacted, 34 responded. Of these, 21 were French-speaking and 13 were Dutch-speaking. The majority were midwives (*n*= 22 versus 3 gynaecologists and 9 from other professions) and had participated in one of the previous phases of the project (*n*= 20). First, the group was surveyed online to elicit parameters without a numerical value. Second, a final workshop was organised with the elicitation group (*n*= 18) to validate or adapt the results of the online survey. If the respondents to the online survey did not agree on the value of a parameter, the value to be assigned to that parameter was discussed. Workshop participants were then asked to vote via an electronic tool to indicate their agreement with the plausibility of the proposed value. If there was no consensus (less than 75% of the voters considered the assigned value to be plausible), a new discussion phase was held, ending with a new vote to reach a consensus on an acceptable value.

Through the process described above (administrative data analysis and elicitation process), an estimate of the evolution of the consumption of midwifery services for different categories of activity was obtained. In order to assess the impact of the different scenarios on the overall demand for midwifery services, it is necessary to aggregate these different trends. However, these activities are often measured in different units (number of services, number of hospital days, etc.), which prevents their aggregation. Therefore, we used an initial allocation key that reflects the relative share of each midwifery activity as observed in 2016. This key was calculated using a mix of survey data (mainly for inpatient care) and administrative claims data (mainly for outpatient services). The impact of changes in the consumption of midwifery services was weighted according to this allocation key.

## Results

### Horizon scanning

From the preparatory online survey, 101 potential factors were suggested for inclusion in the midwifery workforce forecasting model. During the first workshop (*n* = 31 participants), 77 relevant factors that were not yet included in the existing model developed by the Planning Unit, were selected. Detailed results on the retained factors and their potential impact on the future workforce as assessed by the participants are presented in Additional file 1 (see Table 2).

### Narrative scenarios for the future midwifery practice

**Fig. 3:**
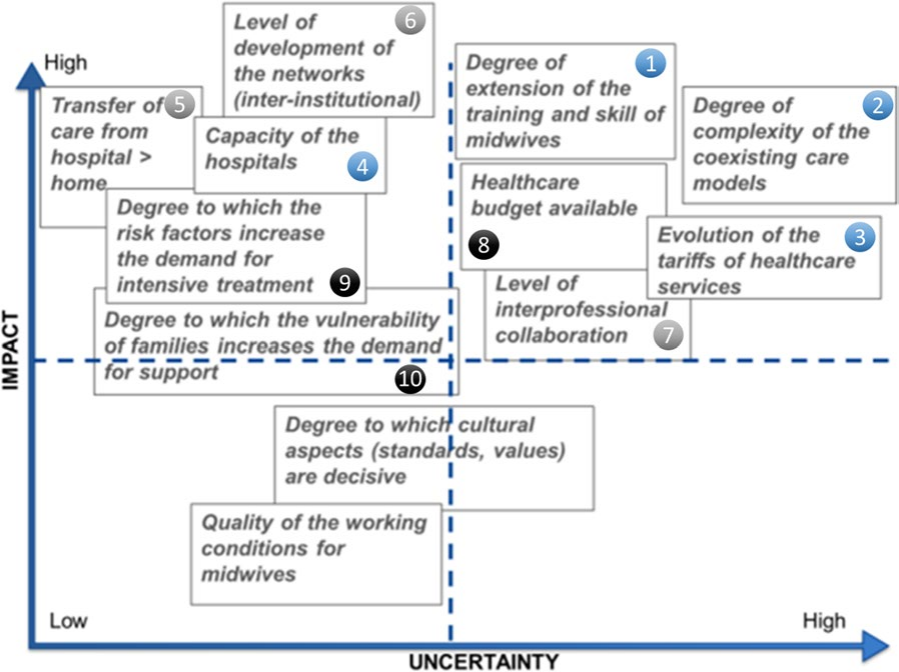
Impact/uncertainty matrix of clustered factors to forecast the future midwifery workforce

Factors identified during the horizon scanning were merged into 12 clusters (see Additional file 1), placed on an impact/uncertainty matrix by the workshop participants (Fig. 3). Ten clusters were positioned above the median line of impact meaning that participants in Workshop 2 (*n*= 24) considered these clusters likely to have a high impact on future midwifery practice. Two other clusters, positioned below the median line of impact, were judged to have a lower impact and were not further considered further in the development of the conceptual framework for narrative scenarios.

The upper right quadrant contains the 5 clusters representing critical uncertainties while the upper left quadrant contains the 5 clusters representing driving forces. Critical uncertainties and driving forces are likely to shape the future of midwifery with varying degrees of probability. It is still possible to simplify the picture by distinguishing 2 underlying meta-clusters, namely the rationale for health care provision (clusters 1–4) and the nature of the networking (clusters 5–7). Contextual factors are independent of the organisation of care but will have a more or less strong impact on supply and demand (clusters 8–10).

**Fig. 4:**
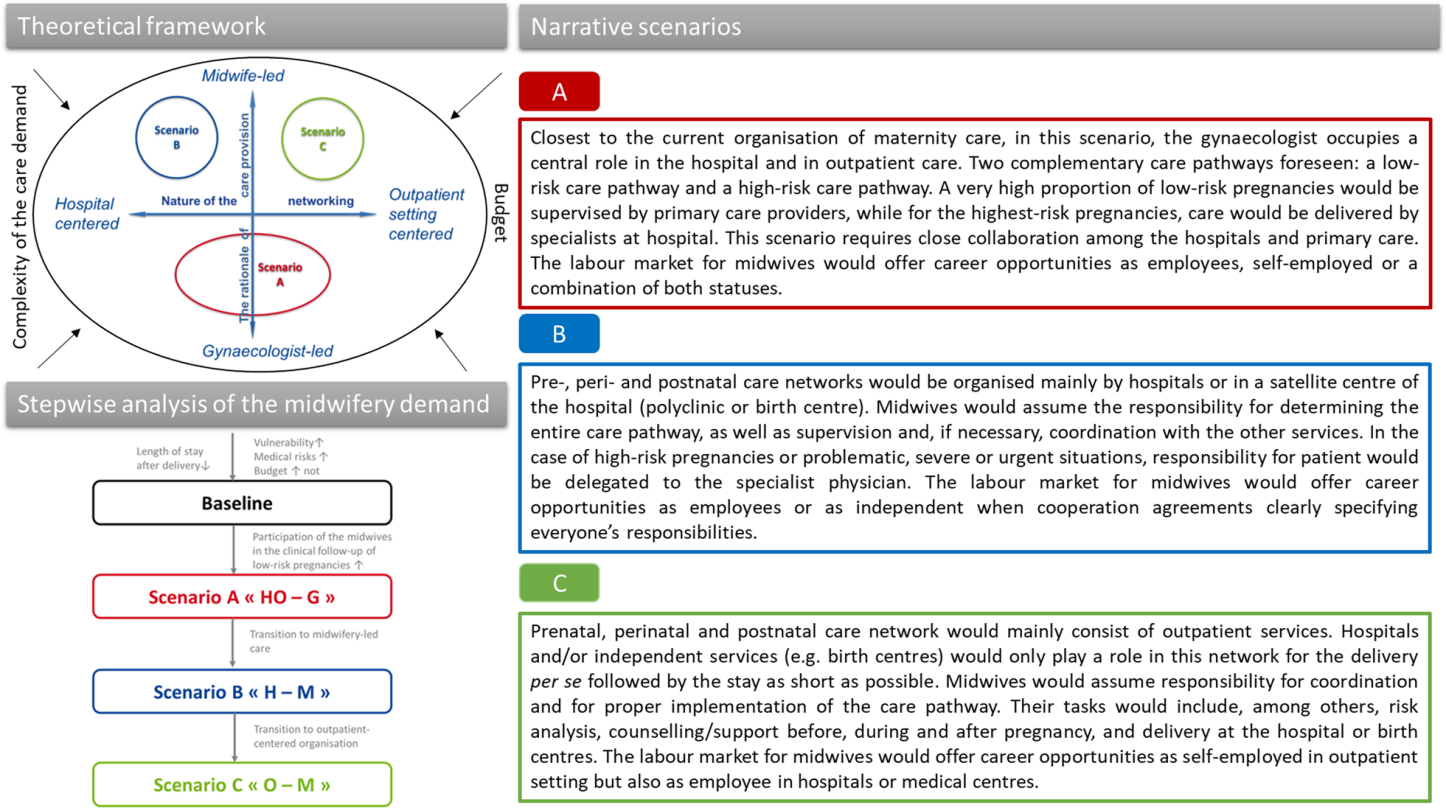
Narrative scenarios based on the theoretical framework derived from the horizon scanning and visualisation of the stepwise analysis on midwifery demand

The framework for the scenarios can be illustrated in Fig. 4.

The first (horizontal) continuum depicts the rationale for care provision, with one extreme representing care provided mainly by midwives (primary care or ‘midwifery-led care’) and the opposite representing care provided mainly by medical specialists (secondary care or ‘specialist-led care’). The second (vertical) continuum illustrates the nature of the networking of perinatal providers around pregnant women and future parents from hospital-centred networks (one extreme) to outpatient networks (the opposite extreme).

The combination of these continua creates a matrix with four quadrants within which all possible alternative scenarios for the future can be positioned alongside a baseline scenario. The baseline scenario takes into account current trends towards a reduction in postnatal length of stay in maternity units and contextual factors (demographic and epidemiological trends, socioeconomic vulnerability and budgetary constraints).

Workshop participants agreed that 3 plausible alternative scenarios were sufficient to predict likely futures. Scenario A is the closest to the current organisation in which the gynaecologist plays a central role, facilitating a close collaboration between the hospital and outpatient networks. In the other two scenarios, care would be provided primarily by midwives, either in a hospital-centred care network (scenario B) or in an ambulatory-centred care network (scenario C). The scenarios are summarised in Fig. 4.

The scenarios illustrate a gradual shift: (1) the transition from the baseline scenario to scenario A was characterised by a greater involvement of midwives in the antenatal care of low-risk pregnancies; (2) the difference between scenario A and B was a shift from a ‘gynaecologist-led-care’ model to a ‘midwifery-led care’ model; and (3) the difference between scenario B and C was the transition from hospital to outpatient care (see Fig. 4).

### Impact of the scenarios on demand for midwifery care

During the elicitation process, 34 professionals, mostly midwives (*n* = 22), gave their input to elicit the parameters of the model, i.e. they were allowed to assign a value to each parameter of the model (see Fig. 2). Their estimates were discussed with 15 midwives in order to reach a consensus on the value assigned to some parameters.

Table 1 shows the values of the parameters that are common to all scenarios, while Table 2 reports the assumptions specific to each scenario. The penultimate column indicates the source of the data.

**Table 1:** Assumptions common to all scenarios

2011	2016	2021	2026	2031	2036	2041	2011–2016 Source	Projection
Number of hospital stays involving childbirth								
125,150	122,458	124,867	126,487	125,726	127,864	129,434	[10, 13]	Until 2025: [10, 13], then constant length of stay and population forecast (Planning Bureau) [14]
Proportion of moderate- to high-risk pregnancies								
17.5%	23.3%	28.7%	34.1%	34.1%	34.1%	34.1%	IMA-AIM	Linear until 2026 then constant
Proportion of the population living in a household with an equivalent disposable income below the poverty threshold								
15.3%	15.9%	16.0%	16.4%	16.4%	16.4%	16.4%	IWEPS [15] on basis of EU-SILC [16]	Linear until 2026 then constant
Proportion of multiparous pregnancies								
55.2%	56.3%	58.4%	60.0%	60.0%	60.0%	60.0%	CEpiP [17, 18] and SPE [19]	Linear until 2026 then constant
Length of the first individual obstetrical session (all risk levels)								
52 min							Elicitation	Constant
Length of the following individual obstetrical sessions for low-risk pregnancies								
30 min							Elicitation	Constant
Length of the following individual obstetrical sessions for moderate- to high-risk pregnancies (including sessions specific to at-risk pregnancies)								
35 min							Elicitation	Constant
Additional length of the individual obstetrical sessions in the event of socioeconomic vulnerability								
15 min							Elicitation	Constant
Average number of additional sessions of preparation for birth and parenthood education in the case of socioeconomic vulnerability								
		1.0	2.0	2.0	2.0	2.0	Elicitation	Elicitation
Length of the individual sessions of birth preparation and parenthood education								
60 min							Elicitation	Constant
Length of the group sessions of birth preparation and parenthood education								
120 min							Elicitation	Constant
Average number of participants in the group sessions								
7							Elicitation	Elicitation
Weighting coefficient applied to the days of hospitalisation (severity level 1)								
Day 1: 155; day 2: 129; day 3: 92; day 4: 84; day 5 and following: 77							ULB, School of Public Health (PACHA) [20]	Constant
Weighting coefficient applied to the days of hospitalisation (severity level 2)								
Day 1: 140; day 2: 144; day 3: 97; day 4: 86; day 5 and following: 82							ULB, School of Public Health (PACHA) [20]	Constant
Weighting coefficient applied to the days of hospitalisation (severity levels 3 and 4)								
Day 1: 172; day 2: 156; day 3: 118; day 4: 108; day 5 and following: 105							ULB, School of Public Health (PACHA) [20]	Constant
Average number of postnatal follow-up and care services until the 5th day postpartum								
0.3	0.8	1/day					Econodat and IMA-AIM	Elicitation
Average number of postnatal follow-up and care services due to complication								
0.1	0.1	0.1					Econodat and IMA-AIM	Constant
Average number of breastfeeding consultations								
0.2	0.4	0.4					Econodat and IMA-AIM	Constant

*IMA-AIM* Intermutualistic Agency grouping all Belgian sickness funds

Econodat is a database reporting the number of all billing codes by year

**Table 2:** Assumptions specific to each scenario

	2011	2016	2021	2026	2031	2036	2041	2011–2016 Source	Projection
Average number of individual obstetrical sessions per pregnancy (low-risk)	1.0	1.4						IMA-AIM	
• Baseline			1.4	1.4	1.4	1.4	1.4		Constant
• Scenario A multiparous			1.6	1.8	2.2	2.6	3.0		Elicitation
Primiparous			1.8	2.2	3.0	3.4	5.0		Elicitation
• Scenarios B and C multiparous			1.9	2.4	3.4	4.5	6.0		Elicitation
Primiparous			2.0	2.6	4.0	5.7	8.0		Elicitation
Average number of individual obstetrical sessions per pregnancy (moderate- to high-risk)	0.8	3.4						Econodat and IMA-AIM	
• Baseline and Scenario A			3.4	3.4	3.4	3.4	3.4		Constant
• Scenarios B and C			3.0	2.7	2.3	1.9	1.6		Elicitation
Average number of individual sessions on preparation for birth and parenthood education per pregnancy	0.3	0.6						Econodat and IMA-AIM	
• Baseline			0.6	0.6	0.6	0.6	0.6		Constant
• Scenarios A, B and C – Multiparous			0.7	0.7	0.8	0.9	1.0		Elicitation
– Primiparous			0.8	1.1	1.6	2.2	3.0		Elicitation
Average number of group sessions of preparation for birth and parenting per pregnancy	0.3	0.4						Econodat and IMA-AIM	
• Baseline			0.4	0.3	0.4	0.4	0.4		Constant
• Scenarios A, B and C – Multiparous			0.2	0.2	0.2	0.2	0.2		Elicitation
– Primiparous			0.5	0.6	0.7	0.8	0.9		Elicitation
Average length of stay (number of days)								[10, 13]	
• Baseline, scenario A and scenario B—examples									
o *APR-DRG 540 (caesarean) – SOI 1 – 25–34 y*	5.7	5.0	4.4	4.0	4.0	4.0	4.0		Until 2025: [10, 13] then constant
*– 35–39 y*	5.7	5.1	4.5	4.1	4.1	4.1	4.1		
o *APR-DRG 560 (vaginal) – SOI 1 – 25–34 y*	4.1	3.7	3.3	3.0	3.0	3.0	3.0		
*– 35–39 y*	4.0	3.6	3.3	3.0	3.0	3.0	3.0		
• Scenario C									
o *APR-DRG 560 – SOI 1: for all age groups*			3.0	2.0	2.0	2.0	2.0		Until 2025: [10, 13] then reduced
*– SOI 2: for all age groups*			3.7	2.5	2.5	2.5	2.5		
o *APR-DRG 540 – SOI 1: for all age groups*			4.4	3.0	3.0	3.0	3.0		
*– SOI 2: for all age groups*			5.4	4.0	4.0	4.0	4.0		
Average number of postnatal monitoring and care services as of the 6th day postpartum	0.9	1.7						Econodat and IMA-AIM	
Baseline, Scenario A, and Scenario B - Multiparous			2.0	2.0	2.0	2.0	2.0		Elicitation
- Primiparous			4.0	4.0	4.0	4.0	4.0		Elicitation
Scenario C - Multiparous			4.0	4.0	4.0	4.0	4.0		Elicitation
- Primiparous			7.0	7.0	7.0	7.0	7.0		Elicitation
Inclusion of coordination tasks via an increase in the length of the prenatal and postnatal consultations (%)		20%						Elicitation	
Baseline and scenario A			20%	20%	20%	20%	20%		Elicitation
Scenarios B and C			21%	22%	23%	24%	25%		Elicitation

*APR-DRG* all patient refined diagnosis related groups, *SOI* severity of illness, *IMA-AIM* Intermutualistic Agency grouping all Belgian sickness funds

Econodat is a database reporting the number of all billing codes by year

### Initial allocation key

During the elicitation process, 94% of the participants estimated that 80% of the midwifery services were provided in hospitals and 20% in outpatient settings (Table 3). Using data on the workforce distribution within hospitals, we estimated that 60% of hospital midwifery services were devoted to postnatal care and 40% to other activities. In outpatient settings, the relative proportions of midwifery services were calculated using the annual number of sessions and the duration of midwifery sessions (Table 1).

**Table 3:** Initial allocation key of midwifery services between hospitals and outpatient settings

	2016 (%)
Outpatient settings	20.0
Prenatal follow-up	5.4
Preparation for birth and parenting	2.7
Postnatal follow-up	11.9
Hospital setting	80.0
Postpartum follow-up	48.0
Other activities	32.0

Given these assumptions, the expected variation in consumption is shown for the different scenarios in Table 4.

**Table 4:** Expected variation in consumption of midwife services compared to 2016 (by 5 years until 2041)

	2021 (%)	2026 (%)	2031 (%)	2036 (%)	2041 (%)
Baseline	+ 5.9	+ 7.1	+ 6.4	+ 8.2	+ 9.6
Scenario A	+ 7.9	+ 11.4	+ 12.3	+ 16.3	+ 20.5
Scenario B	+ 8.1	+ 12.0	+ 13.6	+ 18.7	+ 24.6
Scenario C	+ 17.6	+ 17.4	+ 19.0	+ 24.3	+ 30.4

Between 2016 and 2026, the baseline scenario predicts an increase of 7.1% in the demand for midwifery activities, mainly driven by increasing activities in outpatient postnatal care activities (+ 8.6%), offsetting a 4.6% decrease in inpatient postnatal care activities (due to shorter hospital stays).

Over the same period, the implementation of Scenario A would lead to an increase of 11.4% in midwifery activities. This increase is mainly related to childbirth preparation and parenting education (+ 5.1%) and to a lesser extent to prenatal activities (+ 1.3%).

The transition from Scenario A to Scenario B would have a very small increase related to antenatal and postnatal care (+ 0.3 point of percentage for each activity between the two scenarios).

However, the transition from Scenario B to Scenario C would involve a major organisational change from a hospital-centred model to an outpatient-centred model. This would be reflected in a significant reduction in the length of stay in the maternity ward and an increase in the use of outpatient postnatal services. Scenario C show a 17.4% increase in demand for midwifery activities between 2016 and 2026. This is mainly due to a very strong increase in outpatient postnatal activities (+ 21.6%), partly compensated by a decrease in inpatient postnatal activities (− 12.0%).

## Discussion

### From the baseline scenario to alternative scenarios

The scenario originally developed by the Planning Unit to forecast the midwifery workforce did not initially consider possible, likely, or plausible potential deviations in demand from historical demographic trends [8]. The Robust Workforce Planning Framework led us to develop alternative scenarios to better capture the future demand for midwives, taking into account epidemiological, organisational, technological, economic, environmental, political, social, and ethical influences on future workforce development. Depending on the scenario considered, we estimated an increase of between 11 and 17% in the demand for midwives in 2026 compared with 2016. However, the robustness of the projections depends on several factors such as the quality and completeness of the administrative data, the reliability of the elicitation, the appropriateness of the proxies used, the likelihood of the forecast assumptions and the time horizon of the forecast.

As administrative data are not collected for the purpose of HRH planning, proxies are often used to capture the necessary parameters. For example, care consumption has been used as a proxy for care needs. However, it only represents the expressed demand and does not provide information on unmet need or overuse. In addition, some of the data needed to quantify the scenarios are not available in the administrative databases. Therefore, this was compensated by an elicitation process organised in two steps: (1) a survey sent to 74 stakeholders with a response rate of 47% (including 3 gynaecologists, 22 midwives and 9 professionals from institutions dealing with mother and child care or HRH planning); and (2) a workshop to discuss the survey results with 14 midwives and 1 gynaecologist. The elicitation process was based entirely on the opinions of the experts interviewed. The quality of the results, therefore, depends on the ability of the experts consulted to anticipate the future.

The determination of the allocation key by the elicitation step is a critical feature of the model. Variation in estimation could significantly alter the results obtained. However, an independent check by the midwifery working group of the Planning Unit on their own databases confirms the reported values [8]. A regular monitoring of all elicited parameters would be necessary to ensure the accuracy of the estimates or to integrate deviations. In the future, a prospective collection of missing data could be envisaged to increase the reliability of the estimated parameters.

In Belgium, the Planning Commission produces forecasts with a 25-year horizon. However, the estimation of the future value of some parameters is based on short(er) time series, which means that the degree of uncertainty increases with time. Some authors therefore recommend limiting the forecast period to 10 years [21].

### Plausibility of the scenarios

The scenarios described by the stakeholders and experts in the field favoured a greater involvement of midwives in maternity care, from the antenatal period to the postnatal period, especially for pregnant women with a low risk of complications. This greater involvement would be accompanied by varying degrees of practice autonomy, depending on the scenario. The likelihood of each scenario depends on the time horizon envisaged and many other factors, such as the political will and incentives to reorganise antenatal care and their preference for a particular scenario, the propensity of the system to ensure a more intensive and rapid shift to outpatient care, and access to specialists reserved for high-risk clinical situations. In the near future (5 to 10 years), scenario A is indeed the most likely. However, if we consider a longer time horizon (20 to 25 years), the relevance of the other two scenarios becomes equally likely. In fact, they follow an international trend towards more patient-centred care provided by first-line professionals (midwives and general practitioners) [22]. Such changes usually take several years to implement in daily practice and require adequate training of the professionals concerned. Four years after this study, the Federal Minister of Health's policy note (2023) shows a willingness to invest in the training of midwives and to improve multidisciplinary coordination in the outpatient care of pregnant women and women in labour.

The generation of scenarios is essentially based on the collection and analysis of proposals from midwifery experts in Belgium. It cannot be excluded that a group composed of different experts would have led to different scenarios. The low participation of gynaecologists in the process (despite repeated invitations) and the decrease in the number of participants in the workshops (29, 24, 19, 18 and 15, respectively, for workshops 1 to 5) probably influenced the forms of care proposed for the future. Therefore, this type of exercise needs to be monitored regularly to ensure that the pathways identified for the future remain plausible in the light of legislative, contextual, epidemiological, social, and scientific developments. Forecasts of the HRH workforce, based on a similar methodology and involving stakeholder groups of comparable size, have been developed in the Netherlands [23], The United Kingdom [24, 25], Portugal [26] and Australia [27–29], of which three models concerned midwives. In the Netherlands [23], the training needs of midwives were predicted in 3 steps, combining interviews with 9 experts, 2 workshops with 21 participants to elaborate scenarios and a quantification process based on statistical data and elicitation. Four scenarios were developed on the basis of a theoretical framework presented in 2 dimensions: (1) a continuum from no to a greater shift of tasks from gynaecologists and general practitioners to midwives; and (2) a continuum from no to full collaboration between care providers in primary and specialised care. The more conservative scenario showed a 21% reduction in the number of midwives to be trained over 10 years. The opposite scenario (i.e. greater delegation of tasks to midwives, coupled with the removal of the barrier between primary and specialist care) predicted a 31% increase in the number of midwives to be trained. The UK [25] and Australia [28] focused their scenarios on the supply side of the forecasting model whereas the demand forecast was only based on projected birth rates. The reflection carried out in this study goes beyond the question of HRH alone and raises the question of the future organisation of mother and child care. This strategic positioning depends on the political choices made.

Finally, subjecting the various scenarios to the critical opinion of decision-makers and users of the health system could confirm or undermine their plausibility and, if necessary, make some corrections. Other factors could be taken into account, such as the accessibility of care, the risk of two-tier medicine when moving from one scenario to another, or the quality of care for low-risk pregnancies (caesarean section rates, etc.) [30].

### Impacts of this study on forecasting models in HRH

The Planning Unit used the results of this study to enrich the debate in the Planning Commission. Over a 10-year horizon, the density of midwives (expressed in FTE weighted by the population’s consumption of care by age category—see above) could increase from 13 to 37%, depending on the scenario considered [8].

In addition, the methodology described here is currently being used by the Planning Unit to forecast nurses [31].

## Conclusions

The Robust Workforce Planning Framework combines qualitative methods (prospective analysis and scenario generation) with classical modelling of quantitative projections of human resources for health. Applied to the modelling of the midwifery workforce in Belgium, 3 alternative scenarios for forecasting the demand for midwives were drawn. These scenarios attempt to identify possible deviations from past trends. An increase of between 11 and 17% in the demand for midwives in 2026 compared with 2016 is postulated.

The Robust Workforce Planning Framework provides an opportunity to adjust the HRH workforce modelling and inform decision-makers about the impact of their future decisions on the HRH workforce. In addition, it could be explored how to simultaneously forecast demand for interrelated professions (e.g. midwives and gynaecologists).

### Supplementary Information

**Additional file 1: S1.** Midwifery workforce forecasting model. **S2. **Survey. **S3.** Participants to the workshops.

## Data Availability

Additional supporting information may be found online on the KCE website.

## References

[1] Goemaes R, Fomenko Z, Laubach M, De Coen K, Roelens K, Bogaerts A (2022). Perinatale gezondheid in Vlaanderen Jaar 2021.

[2] Leroy C, Van Leeuw V (2022). Santé périnatale en Wallonie–Année 2021.

[3] Van Leeuw V, Leroy C (2022). Santé périnatale en Région bruxelloise–Année 2021.

[4] Planning Unit for Health Professionnal Supply [Cellule Planification de l'Offre des Professions des Soins de Santé] (2022). Statistiques annuelles des professionnels des soins de santé en Belgique - Nombre de professionnels en droit d’exercer au 31/12/2021 et influx 2021.

[5] Durand C, Jouck P, Miermans P, Steinberg P, Vivet V (2022). PlanCad Sages-femmes 2019, Cellule Planification des professions de soins de santé.

[6] Gerkens S, Merkur S: Belgium: health system review. Copenhagen: World Health Organization. European Observatory on Health Systems and Policies Regional Office for Europe; 2020.

[7] Roberfroid D, Leonard C, Stordeur S (2009). Physician supply forecast: better than peering in a crystal ball?. Hum Resour Health.

[8] Durand C, Vivet V, Jouck P, Miermans P, Steinberg P (2020). Scénarios de base de l’évolution de la force de travail Sages-femmes 2017–2042, Cellule Planification des professions de soins de santé, Service Professions des soins de santé et pratique professionnelle, DG Soins de santé.

[9] Willis G, Cave S, Kunc M (2018). Strategic workforce planning in healthcare: a multi-methodology approach. Eur J Oper Res.

[10] Amanatidou E, Butter M, Carabias V, Könnölä T, Leis M, Saritas O, Schaper-Rinkel P, van Rij V (2012). On concepts and methods in horizon scanning: lessons from initiating policy dialogues on emerging issues. Sci Public Policy.

[11] Benahmed N, Hendrickx E, Adriaenssens J, Stordeur S (2016). Health workforce planning and midwifery-specific data. KCE reports.

[12] The Centre for Workforce Intelligence. Horizon scanning. Analysis of key forces and factors. In: Centre for Workforce Intelligence technical paper series No. 0006 UK; 2014.

[13] Van de Voorde C, Van den Heede K, Beguin C, Bouckaert N, Camberlin C, de Bekker P, Defourny N, De Schutter H, Devos C, Gerkens S (2017). Required hospital capacity in 2025 and criteria for rationalisation of complex cancer surgery, radiotherapy and maternity services. KCE Reports.

[14] Bureau Fédéral du Plan. Perspectives de population 2017-2070. 2018.

[15] Institut wallon de l’évaluation, de la prospective et de la statistique (IWEPS), Taux de risque de pauvreté. 2018. Available from https://www.iweps.be/indicateur-statistique/taux-de-risque-de-pauvrete/

[16] Eurostat. EU statistics on income and living conditions (EU-SILC). 2018. Available from https://ec.europa.eu/eurostat/web/microdata/european-union-statistics-on-income-and-living-conditions

[17] Leroy C, Debauche C, Daelemans C, Debiève F, Van Leeuw V. Santé périnatale en Wallonie – Année 2016. Centre d’Épidémiologie Périnatale; 2018.

[18] Van Leeuw V, Debauche C, Daelemans C, Debiève F, Leroy C. Santé périnatale en Région bruxelloise – Année 2016. Centre d’Épidémiologie Périnatale; 2018.

[19] Devlieger R, Martens E, Martens G, Van Mol C, Cammu H. Perinatale Activiteiten in Vlaanderen 2016. Studiecentrum voor Perinatale Epidemiologie; 2017.

[20] Pirson M, Delo C, Martins D, Leclerq P (2012). Evaluation du coût de l’accouchement: perspective hospitalière. Gunaikeia.

[21] Van Greuningen M, Batenburg RS, Van der Velden LF (2013). The accuracy of general practitioner workforce projections. Hum Resour Health.

[22] World Health Organization. WHO recommendations on antenatal care for a positive pregnancy experience. 2016. 28079998

[23] Batenburg R, van der Lee I, Wiegers T, van der Velden L (2013). De arbeidsmarkt voor verloskundigen in 2012 en 2022/2027—Een capaciteitsraming op basis van beleidsrijke scenario’s.

[24] Buchan J, Seccombe I (2012). Using scenarios to assess the future supply of NHS nursing staff in England. Hum Resour Health.

[25] The Centre for Workforce Intelligence. Future midwifery workforce projections-starting the discussion. Future midwifery workforce projections-starting the discussion. Centre for Workforce Intelligence. 2013.

[26] Gregorio J, Cavaco A, Velez Lapao L (2014). A scenario-planning approach to human resources for health: the case of community pharmacists in Portugal. Hum Resour Health.

[27] Crettenden I, McCarty M, Fenech B, Heywood T, Taitz M, Tudman S (2014). How evidence-based workforce planning in Australia is informing policy development in the retention and distribution of the health workforce. Hum Resour Health.

[28] Health Workforce Australia. Health Workforce 2025—doctors, nurses and midwives. Vol 1. Adelaïde; 2012.

[29] Laurence C, Karnon J (2017). What will make a difference? Assessing the impact of policy and non-policy scenarios on estimations of the future GP workforce. Hum Resour Health.

[30] Devos C, Cordon A, Lefèvre M, Obyn C, Renard F, Bouckaert N, Gerkens S, Maertens de Noordhout C, Devleesschauwer B, Haelterman M (2019). Performance of the Belgian health system—report 2019—supplement, KCE reports.

[31] Vivet V, Durand C, Jouck P, Nkenné D, Steinberg P. La force de travail des infirmiers en 2043: projection future sur base de l'influx réel jusque 2020. Résultats des scénarios de base: SYNTHÈSE. Cellule Planification de l’offre des professions des soins de santé. Service Professions de Santé et Pratique professionnelle DG Soins de santé. SPF Santé publique, Sécurité de la chaîne alimentaire et Environnement; 2022.

