# Supplementary material for: Managing uncertainty in forecasting health workforce demand using the Robust Workforce Planning Framework: the example of midwives in Belgium
Source: Hum Resour Health. 2023 Sep 18;21:75. doi: 10.1186/s12960-023-00861-1 (PMC10506318; doi:10.1186/s12960-023-00861-1)
Supplement: Supplementary file 1 — Additional file 1: S1. Midwifery workforce forecasting model. S2. Survey. S3. Participants to the workshops. [file 12960_2023_861_MOESM1_ESM.docx]

supplementary materials

Table of content

[1 midwifery workforce forecasting model 1](#_Toc145492513)

[2 Survey 3](#_Toc145492514)

[2.1 Invitation mail 3](#_Toc145492515)

[2.2 Survey 4](#_Toc145492516)

[2.2.1 Introduction 4](#_Toc145492517)

[2.2.2 Survey 4](#_Toc145492518)

[2.2.3 Closing message 11](#_Toc145492519)

[2.3 List of factors identified during the first workshop according to the domain of analysis factors influencing the system 12](#_Toc145492520)

[2.4 Clustering of factors by the participants 16](#_Toc145492521)

[3 Participants to the workshops 18](#_Toc145492522)

# midwifery workforce forecasting model

Table 1 – Description of the parameters included in the forecasting model for midwives in Belgium

| Parameter | Label |
| --- | --- |
| Belgian Diplomas | Number of graduates trained in Belgium, by nationality and language of qualification, in year t+3 (Flemish Community) or t+4 (French Community)*. |
| Registration rate | Rate of registration with the Cadaster^$^, by nationality and language of qualification, in year t+3 (Flemish Community) or t+4 (French Community)*. |
| Registration Cadaster^$^ | Number of professionals entitled to practice, registered in the Cadaster^$^, by nationality, age and language of qualification, in year t+3 (Flemish Community) or t+4 (French Community)*. |
| Inflow Belgian registration | Entries due to graduates trained in Belgium, by nationality, age and language of qualification, in year t+3 (Flemish Community) or t+4 (French Community)*. |
| Inflow professionals NonBel | Entries due to graduates trained abroad, by nationality, age and language community, in year t+3 (Flemish Community) or t+4 (French Community)*. |
| Total Inflow | Total entries, by nationality, age and host language community in year t+3 (Flemish Community) or t+4 (French Community)* |
| Existing Stock | Stock of professionals in year t, by nationality, age and language community |
| Survival rate | Survival rate, by age and language community |
| Stock year+5 | Stock of professionals in year t+5, by nationality, age and language community |
| Participation & sector repartition rate | Participation rate according to sector of activity (health sector versus other sectors) and within the health sector, according to nationality, age and language community |
| Inactive individuals | Number of inactive individuals |
| Individuals in Workforce | Number of active individuals, by sector of activity, nationality, age and language community |
| Activity rate | Activity rate, by sector of activity, nationality, age and language community. The activity rate takes into account   1. the reduction in full-time equivalents (FTE) for employees aged 45 and over working in a hospital to reflect the days off work (45-49 years: 1 day off / month, 50-54 years: 2 days / month and 55 years and over: 3 days / month maximum for full-time work). 2. A gradual increase in the legal retirement age (from 2025: 66 years, from 2030: 67 years), applied to the participation rate, the breakdown between sectors of activity and the calculation of FTEs. 3. The fact that, from 1 October 2018, newly qualified midwives will no longer be able to carry out nursing activities outside the field specific to midwives. |
| FTE in Workforce | Number of full-time equivalents in the workforce by nationality, age and language community |
| Female Belgian Population | Belgian female population of childbearing age (15 - 49) by age and linguistic community |
| Care consumption rate | Care consumption rates by age and language community |
| Weighted Population | Population adjusted by care consumption measured in terms of expenditure payable by the National Institute for Health and Disability Insurance, according to age and language community |
| Crude densities | Density of active professionals |
| Weighted densities | Density of active professionals weighted by consumption adjusted by age and linguistic community |

* The duration of the basic training of a midwife is currently 3 years in Dutch speaking community and 4 years in French-speaking community.

$ Cadaster: official registry of the Federal Health Authorities including all health professional entitled to practice in Belgium

# Survey

## Invitation mail

Dear,

KCE has been commissioned by the Ministry of Public Health Food Chain Safety and Environment (Health Professionals Planning Unit) to investigate the factors likely to influence the future midwifery workforce and its professional practice.

This study consists of two parts:

- Horizon scanning of factors influencing the midwifery workforce
- Scenario development, including:
  - Developing and describing of narrative scenarios
  - Quantifying the supply and the demand of midwives in each of the selected scenarios

The survey below has been developed to respond to the first part of the study related to the horizon scanning study. The aim is to identify as fully as possible the factors that influence the midwifery workforce, both positively and negatively. These factors are diverse and relate to the organisation of the profession, health services, education, and research, but also to expected demographic trends (including fertility and birth rates), etc.

The potential impact of technology, the economy, the environment, politics, social issues and ethics on the future workforce is all issues also addressed in the questionnaire.

As no one is an expert in all these areas, you have been selected from a wide range of experts in a wide range of fields. We, therefore, ask you to answer only those questions that fall within your own area of expertise (demography, economics, midwifery, obstetrics, care planning, etc.).

The list of factors identified through this questionnaire will then be discussed and prioritised in working groups with the participants in this survey.

The second part of the research (scenario generation) will be based on the results of this questionnaire and the working groups.

Thank you for your participation and please feel free to contact us for any further information.

## Survey

### Introduction

Welcome to the survey on Horizon scanning – Midwives

Horizon scanning explores the potential challenges, opportunities, and future developments in the future that may influence midwifery workforce planning. This includes the influence of technological, economic, environmental, political, social, and ethical factors on the future of the workforce under consideration.

The questionnaire is structured so that you can respond to the topics on which your expertise is sought. You are free to skip sections or answer only some of the questions in a section.

You can pause the questionnaire at any point by clicking on 'Resume Later' before leaving the questionnaire. This will save your answers when you return to the questionnaire.

If you encounter any technical problems, please do not hesitate to contact us at the following address: XXX. The survey will close at midnight on 18 March.

### Survey^[[1]](#footnote-1)^

**Section 1: Economy**

This is the economic system in which the health and social care system functions. This includes global economic influences, national finance, GDP, work and reward, monetary systems, health and social care budgets, funding, and affordability.

In this section, we have already identified the following factors that are likely to influence the future supply and/or demand of midwives

- Reduction in the growth norm in the health budget
- Rationalisation of the hospital sector by closing beds and/or maternity units
- Impoverishment of the population, increase in the number of people in social and economic vulnerability (poverty, migration).

In your opinion, which factors should complete this list?

- I have no expertise in this area
- I would like to add:

Submit

**Section 2: Environment**

The theme involves the state of the natural environment. This incorporates the quality of the natural environment, climate change, water availability and quality, food, and agriculture.

In this section, we have already identified the following factors as likely to influence the future supply and/or demand of midwives

- Environmental impacts on reproductive health (e.g. reduced fertility due to pollution).

In your opinion, which factors should complete this list?

- I have no expertise in this area
- I would like to add:

Submit

**Section 3: Population**

This includes population characteristics such as age composition, gender ratios and regional distributions, and the drivers of the population change such as births, deaths and migration.

In this section, we have already identified the following factors that are likely to influence the future supply and/or demand of midwives

- Trend in the number of births
- Trend in the number of births with mother aged over 40
- Trend in the number of births with mother aged under 18
- Trend in the number of multiple births
- Trend in the number of large parity
- Trend in the number of births at < 28 weeks' gestation

In your opinion, which factors should complete this list?

- I have no expertise in this area
- I would like to add:

Submit**Section 4: Society, culture and behaviour**

Social community, social behaviour and public attitudes are involved in this factor theme. It includes ethical norms, quality of life, expectations of the health and social care system, and the extent at which the public is empowered. It also includes interpersonal relationships, social support networks, community engagement, health behaviours, public health education and prevention, and education.

In this section, we have already identified the following factors as likely to influence the future supply of and/or demand for midwives

- Mother’s level of education
- Trend in the number of single-parent families families
- Mothers' preferences for medical or non-medical management of delivery
- Cultural practices and cultural differences in choice of provider (gynaecologist, general practitioner, midwife, female/male provider, etc.).

In your opinion, which factors should complete this list?

- I have no expertise in this area
- I would like to add:

Submit

**Section 5: Health and well-being**

Health and well-being of the population are under this factor theme, including the demand for both health care and social care and support.

In this section, we have already identified the following factors as likely to influence the future supply and/or demand for midwives

- Birth presentation (e.g. breech, transverse or oblique lie)
- Maternal biomedical characteristics (obesity, chronic conditions such as diabetes, hypertension, etc.)
- Maternal mental health (depression, pregnancy denial, addiction, domestic violence, etc.).

In your opinion, which factors should complete this list?

- I have no expertise in this area
- I would like to add:

Submit

**Section 6: Policies and legislation****^[[2]](#footnote-2)^**

This is the political framework in which the health and social care system operates. National and regional legislative frameworks are examined, as well as the influence of European and international policy decisions.

In this section, we have already identified the following factors as likely to influence the future supply and/or demand of midwives

- Reduction in length of hospital stay

In your opinion, which factors should complete this list?

- I have no expertise in this area
- I would like to add:

Submit

**Section 7: Research and technology**

Technology and its application in the health and social care system include technology development, innovation and diffusion, and the impacts on service delivery, availability, outcomes, and costs.

In this section, we have already identified the following factors that are likely to influence the future supply and/or demand of midwives

- Use of portable neonatal screening tests
- Use of ultrasound by midwives

In your opinion, which factors should complete this list?

- I have no expertise in this area
- I would like to add:

Submit

**Section 8: Employment and the labour market**

The labour market external to the health and social care workforce incorporates employment trends, industry competition for labour, and employee rights. It considers the wider non-health and non-social care workforce availability and ability (skills and competences).

In this section, we have already identified the following factors as likely to influence the future supply and/or demand of midwives

- Transition of the postnatal period from hospital to home
- Potential substitution from obstetricians to midwives (with master’s degree or not)
- Creation of the postnatal assistant profession
- Effective retirement age, taking into account early retirement
- End of career arrangements
- Deferring the retirement age (at 67 years)
- Freed movement of midwives within the EU

In your opinion, which factors should complete this list?

- I have no expertise in this area
- I would like to add:

Submit

**Section 9: Services**

This theme entails the services delivered to service users to meet a need. All services have a value to users, which can only be defined by user. This theme includes not just the cost of the service (if any) but the efforts involves (e.g. waiting time, distance to travel, or ease of use).

In this section, we have already identified the following factors as likely to influence the future supply and/or demand of midwives

- Trend in the workforce of gynaecologists and GP
- Legal Exclusion of midwives from working as nurses, restricted to midwifery from 2018.

In your opinion, which factors should complete this list?

- I have no expertise in this area
- I would like to add:

Submit

**Section 10: Delivery model**

This is the care delivery model by which the workforce and supporting infrastructure are applied in the delivery of health and social care. It includes aspects such as where the care is delivered (integrated care, shift of care), how the care is to be delivered (skill mix), governance, ethical practice guidelines, and the legislative framework (including standards and regulation, safety requirements, and the ethical framework) in which care is delivered. The care delivery model also includes the split between public and private sector delivery and the acceptance of health tourism.

Among the factors likely to influence the future supply and/or demand of midwives, we have already identified in this section the following:

- Access to medically assisted reproduction for European or foreign patients
- Antenatal care provided by midwives (exclusively) or by other professionals or as part of a shared care model
- Antenatal care in the form of individual consultations (one woman at a time) or group consultations (groups of 6-8 women during the same consultation).

In your opinion, which factors should complete this list?

- I have no expertise in this area
- I would like to add:

Submit

**Section 11: Workforce ^b^**

The theme involves the workforce applied in the delivery of health care and social care. It includes characteristics such as workforce size, skill, productivity, recruitment, attrition, and training. More qualitative factors, such as the attractiveness of the profession, the expectations of professionals, the satisfaction of professionals, and early departures or returns to the profession.

In this section, we have already identified the following factors as likely to influence the future supply and/or demand of midwives

- Number of midwives licensed to practice
- Number of midwives working in the healthcare system
- Number of FTE hospital midwives
- Number of FTE self-employed midwives

In your opinion, which factors should complete this list?

- I have no expertise in this area
- I would like to add:

Submit

**Section 12: Workforce training and education**

This theme involves the training and education of the workforce, including formal training and progression to qualification, and continuing professional development while in employment.

In this section, we have already identified the following factors as likely to influence the future supply and/or demand of midwives

- Creation of the maternity assistant profession for the postnatal period
- Extension of midwifery training duration to 4 years (Flanders)
- Extension of midwifery training duration at the national level to master level
- Recognition of new skills for midwives (e.g. ultrasound scans during pregnancy, participation in genetic counselling)
- Extension of the legal list of midwifery procedures (with or without prescription/medical supervision).

In your opinion, which factors should complete this list?

- I have no expertise in this area
- I would like to add:

Submit

**Section 13: Resources and Infrastructure ^b^**

The resources and infrastructure applied in the delivery of health and social care includes infrastructure (such as hospitals) and resources (e.g. electricity, water, etc.) on the one hand, and services (e.g. health care) on the other.

Among the factors likely to influence future supply and/or demand for midwives, we have already identified in this section the following

- The need for human resources to ensure the transition of postnatal care from hospital to home
- The need for material resources to ensure the transition of postnatal care from hospital to home
- Reduced hospital capacity following the transition of postnatal care from hospital to home.

In your opinion, which factors should complete this list?

- I have no expertise in this area
- I would like to add:

Submit

**Section 14: Service users**

End-users of services and delivery of care to the service users (for example patients and users of social care services) are included in this theme. The act of delivering care will result in workload, quality of care, and public experiences of care.

In this section, we have already identified the following as factors likely to influence future supply and/or demand for midwives

- Maintaining quality of care and low rates of (maternal and infant) adverse events
- Parental satisfaction with antenatal and postnatal care
- Quality indicators (breastfeeding, satisfaction, postnatal depression)

In your opinion, which factors should complete this list?

- I have no expertise in this area
- I would like to add:

Submit

### Closing message

Thank you very much for your participation.

The workshops will take place during the week of 26 March at the KCE (55 Boulevard du Jardin Botanique, 1000 Brussels). If you haven't already done so, don't forget to fill in the doodle to take part in the working groups (https://doodle.com/poll/t6u6s5unemzdihrm). This meeting is considered as an expert meeting. Your participation will therefore be financially compensated.

## List of factors identified during the first workshop according to the domain of analysis factors influencing the system

Table 2 - Classification of factors by area and the score of relevance to the midwifery workforce forecasting

| N° | Wording of factors | Score of relevance | Domain |
| --- | --- | --- | --- |
| 1 | Economic vulnerability in pregnant women | High | Economy |
| 2 | Transition at the national level from bachelor’s degree (3 or 4 years) to master’s degree (5 years) for the midwifery training | High | Workforce training and education |
| 3 | Evolution of the legal competence of midwives | High | Delivery model |
| 4 | Gynaecological follow-up of non-pregnant women by midwives | High | Delivery model |
| 5 | Organisation of care based on midwifery-led care | High | Delivery model |
| 6 | One-to-one care organisation | High | Delivery model |
| 7 | Creation of a nomenclature code for perineal re-education | High | Politics and legislation |
| 8 | Creation of a code for preconception consultations | High | Politics and legislation |
| 9 | Use and development of Evidence Based Practice by midwives | High | Research and technology |
| 10 | Number of pregnant women with diabetes | High | Health and well-being |
| 11 | Number of primiparous women | High | Health and well-being |
| 12 | Number of multiparous women (>1 & < 4) | High | Health and well-being |
| 13 | Number of large multiparous women (>4) | High | Health and well-being |
| 14 | Number of antenatal depressions | High | Health and well-being |
| 15 | Number of postnatal depressions | High | Health and well-being |
| 16 | Number of obese pregnant women | High | Health and well-being |
| 17 | Maternal morbidity | High | Health and well-being |
| 18 | Creation, approval and funding of new midwifery-led unit in and outside of hospitals | High | Services |
| 19 | Mother's preference for midwifery care | High | Society, culture, and behaviour |
| 20 | Mother's preference for care provided by gynaecologist | High | Society, culture, and behaviour |
| 21 | Need for health education for future parents | High | Society, culture, and behaviour. |
| 22 | Creation of the maternity assistant profession for the postnatal period | High | Society, culture, and behaviour |
| 23 | Rate of readmission, of newborn babies | High | Service users |
| 24 | Readmission rate for mothers | High | Service users |
| 25 | Parental satisfaction with prenatal, perinatal and postnatal care | High | Service users |
| 26 | Rate of social vulnerability among pregnant women | Medium | Economy |
| 27 | Prolongation of midwifery training to 4 years (Flanders) | Medium | Workforce training and education |
| 28 | Certification of professional quality | Medium | Workforce training and education |
| 29 | Legal obligation to be on call | Medium | Delivery model |
| 30 | Organisation of care based on the Centering Pregnancy model | Medium | Delivery model |
| 31 | Organisation of postnatal care in a multidisciplinary network | Medium | Delivery model |
| 32 | Shorter hospital stays after childbirth | Medium | Politics and legislation |
| 33 | Length of parental leave for working midwives | Medium | Politics and legislation |
| 34 | Creation of an integrated primary care policy | Medium | Politics and legislation |
| 35 | Perinatal mortality rate | Medium | Population |
| 36 | Number of pregnant women in rural areas | Medium | Population |
| 37 | Number of late pregnancies (> 40 years) | Medium | Population |
| 38 | Number of women of immigrant background (cultural differences, health behaviour, etc.) | Medium | Population |
| 39 | Number of fertility tests | Medium | Health and well-being |
| 40 | Change in the number of pregnant women with diabetes due to a change in the detection threshold | Medium | Health and well-being |
| 41 | Number of pregnant women with drug addictions | Medium | Health and well-being |
| 42 | Number of pregnant women with alcohol addictions | Medium | Health and well-being |
| 43 | Number of caesarean sections | Medium | Health and well-being |
| 44 | Number of premature births (excluding multiple pregnancies) | Medium | Health and well-being |
| 45 | Neonatal morbidity rate | Medium | Health and well-being |
| 46 | Number of breastfeeding mothers | Medium | Health and well-being |
| 47 | Density of general practitioners (GP) by geographical area | Medium | Services |
| 48 | Density of gynaecologists by geographical area | Medium | Services |
| 49 | Education level of expectant mothers | Medium | Society, culture, and behaviour |
| 50 | Number of single-parent families | Medium | Society, culture, and behaviour |
| 51 | Number of pregnant women who are victims of domestic violence | Medium | Society, culture, and behaviour |
| 52 | Number of pregnant women not covered by a sickness found | Medium | Society, culture, and behaviour |
| 53 | Number of pregnant women who do not speak French or Dutch | Medium | Society, culture, and behaviour |
| 54 | Growth norm for healthcare budget | Low | Economy |
| 55 | Lump-sum payment for reproductive care covering the entire process, from the preconception phase to the postpartum period | Low | Economy |
| 56 | Strengthening lifelong learning | Low | Workforce training and education |
| 57 | Involvement of midwives in NICU services | Low | Delivery model |
| 58 | Introduction of home phototherapy | Low | Delivery model |
| 59 | Access to medically assisted procreation for European and foreign patients | Low | Delivery model |
| 60 | Privatisation of home postpartum care | Low | Delivery model |
| 61 | Creation of an ultrasound code for midwives | Low | Politics and legislation |
| 62 | Funding of primary care by the federated entities | Low | Politics and legislation |
| 63 | Illegal migration rate | Low | Population |
| 64 | Legal migration rate | Low | Population |
| 65 | Maternal mortality rate | Low | Population |
| 66 | Reduction in hospital capacity (closure of beds and/or maternity units) | Low | Resources and infrastructure |
| 67 | Smoking rate among pregnant women | Low | Health and well-being |
| 68 | Number of pre-implantation diagnostics | Low | Health and well-being |
| 69 | Number of women affected by genital mutilation | Low | Health and well-being |
| 70 | Number of haemoglobinopathies | Low | Health and well-being |
| 71 | Number of multiple pregnancies | Low | Health and well-being |
| 72 | Number of voluntary terminations of pregnancy | Low | Health and well-being |
| 73 | Number of physiological births (without medical intervention) | Low | Health and well-being |
| 74 | Role of midwives within the ONE | Low | Services |
| 75 | Role of midwives within K&G | Low | Services |
| 76 | Impact of working conditions on the health of pregnant women - fetuses | Low | Society, culture, and behaviour |
| 77 | Number of blended families | Low | Society, culture, and behaviour |

## Clustering of factors by the participants

The grouping of factors enabled the identification of 12 clusters:

1. Level to which family vulnerability increases the need for support
2. Socio-economic vulnerability
3. Income level
4. Family composition
5. Cultural context
6. Legal / illegal migration
7. Addiction issues in the family
8. Domestic violence context
9. Level of education and literacy
10. History of depression (before and/or after pregnancy)
11. Level to which the cultural aspects (norms, value) are decisive
12. Future parents’ need for support in the parenting and health areas
13. Parents’ satisfaction with antenatal, peri- and postnatal care
14. Mother’s preference for a gynaecologist or a midwife
15. Choose of breastfeeding
16. Use of pre-implantation diagnostics
17. Primiparous <> multiparous (≈ desire for children)
18. Choosing to deliver without medical assistance
19. Hospital capacity
20. Reduction in hospital capacity (closure of beds and/or maternity units)
21. Reduction of hospital length of stay after delivery
22. Level to which care shifts from hospital to home care
23. Privatisation of home postpartum care
24. Creation, approval and funding of new midwifery-led unit in and outside of hospitals
25. Primary midwifery care
26. Employment opportunity for midwives
27. Midwifery practice in primary care
28. Midwifery practice as at Kind & Gezin, ONE
29. Available budget for health care
30. Growth norm for healthcare budget
31. Funding of primary care by the federated entities
32. Certification of professional quality and its impact on the hospital budget
33. Change in pricing role of healthcare
34. Creation of nomenclature code (official tariff) :
35. Ultrasounds performed by midwives
36. Perineal re-education
37. Preconception consultations
38. Lump-sum payment for reproductive care covering the entire process, from the preconception phase to the postpartum period
39. Complexity degree of coexisting care models
40. Midwifery-led unit
41. Centering Pregnancy
42. One-to-one care
43. Legal obligation to be on call for midwives in primary care
44. Level to which risk factors increased complex care demand
45. Health status : Hypertension, diabetes (new detection threshold), gestational diabetes, haemoglobinopathy
46. Physical status : Age, obesity, addictions (alcohol, tabaco, drugs), genital mutilations
47. History:
48. Cesarean section, one or more prior pregnancy, premature birth, pregnancy termination, prenatal mortality, maternal morbidity
49. Hospital readmission rate for mother or newborn
50. Level of prolongation of training and competence development of midwives
51. Development of midwives’ competences
52. Gynaecological follow-up of non-pregnant women by midwives
53. Recognition of additional skills
54. Participation of midwives in EBM (Evidence-Based Medicine) researches
55. Prolongation and expansion of the midwifery training
56. General expansion of the training offer (especially out-of-hospital training
57. Prolongation of midwifery training to 4 years (Flanders)
58. At the national level, prolongation of midwifery training midwifery master’s degree (5 years)
59. Level of network development (inter-institutions)
60. Organisation of postnatal care in a multidisciplinary network
61. Creation of the maternity assistant profession
62. Creation of an integrated primary care policy
63. Level of interprofessional collaboration
64. Multidisciplinary networking
65. Geographical density of :
66. Gynaecologists
67. General Practitioners
68. Involvement of midwives in NICU services
69. Working conditions for midwives
70. Length of parental leave for working midwives
71. Difficult working condition

# Participants to the workshops

| Profession | Gender | Language | Ws 1 | Ws 2 | Ws 3 | Ws 4 | Ws 5 |
| --- | --- | --- | --- | --- | --- | --- | --- |
| **Health Professionals** | | | | | | | |
| G – PU | F | Fr | x |  |  |  |  |
| G - PU | M | Nl |  |  | x |  | x |
| G - ONE | M | Fr | x |  |  |  |  |
| Mw – PU | F | Nl |  |  | x |  |  |
| Mw – PU | F | Nl | x |  |  |  |  |
| Mw – PM | F | Fr | x | x |  | x |  |
| Mw – PM | F | Fr |  |  |  |  | x |
| Mw – PM | F | Fr |  |  |  |  | x |
| Mw – PM | F | Fr | x | x | x | x | x |
| Mw – PM | F | Fr |  |  | x | x |  |
| Mw – PM | F | Fr | x |  |  |  |  |
| M – PM | F | Fr |  |  |  | x |  |
| Mw – PM | F | Nl | x |  |  |  | x |
| Mw – PM | F | Nl |  |  |  |  | x |
| Mw – PM | F | Nl | x | x |  |  |  |
| Mw – PM | F | Nl |  | x |  |  |  |
| Mw – Health Center | F | Fr |  | x | x |  |  |
| Mw – midwifery–led unit | F | Fr |  |  |  |  | x |
| Mw – head of maternity | F | Nl |  |  |  | x |  |
| Mw – head of maternity | M | Nl | x |  |  |  |  |
| Mw – head of maternity | F | Fr |  | x | x | x | x |
| Mw – head of maternity | F | Fr |  |  |  | x |  |
| Nurse – head of maternity | F | Fr | x | x | x | x |  |
| Mw – Maternity | F | Nl |  | x | x |  | x |
| Mw – Maternity | F | Nl |  | x | x | x |  |
| Mw – Maternity | M | Nl |  | x |  |  | x |
| Mw – Maternity | F | Fr |  | x | x | x | x |
| Mw – Maternity | F | Fr |  | x | x | x |  |
| K&G | F | Nl |  | x | x |  |  |
| Mw – ONE | F | Fr | x | x |  |  |  |
| Social worker– ONE | F | Fr |  | x | x | x |  |
| Social worker– ONE | F | Fr |  | x |  |  |  |
| Director ONE | F | Fr | x |  |  |  |  |
| Medical center federation – lifelong training | F | Fr | x |  |  |  |  |
| Pediatrician | F | Fr | x |  |  |  |  |
| **Authorities** |  |  |  |  |  |  |  |
| Mw – Federal health authority | F | Nl | x |  |  |  |  |
| Walloon health authority | M | Fr | x | x | x | x |  |
| Flemish health authority | F | Nl | x |  |  |  |  |
| Mw – National Council of Midwifery | F | Nl |  | x |  |  |  |
| Pediatrician – Mother & Child Council | F | Fr | x |  |  |  |  |
| Planning Unit | F | Fr | x |  |  |  |  |
| Planning Unit | F | Fr | x | x | x | x |  |
| Planning Unit | F | Nl | x | x | x | x |  |
| STATBEL | M | Fr | x |  |  |  |  |
| **Professional organisations** | | | | | | | |
| Association of health institutions in Wallonia and Brussels | F | Fr | x |  |  |  |  |
| Professional association of midwives – Mw | F | Fr | x | x | x | x |  |
| Professional association of midwives – Mw | F | Fr | x |  |  |  | x |
| Professional association of midwives – Mw | F | Fr | x | x | x |  | x |
| Professional association of midwives – Mw | F | Fr |  | x | x | x | x |
| Professional association of midwives – Mw | F | Nl | x |  |  |  | x |
| Professional association of midwives – Mw | F | Nl |  | x |  | x |  |
| G –Professional association of gynaecologists | M | Nl | x |  |  |  |  |
| G –Professional association of gynaecologists | M | Nl | x |  |  |  |  |

Ws: Workshop; G: Gynaecologist; Mw: Midwife; PU: Professor University; PM: Professor Midwifery school; PF: representative of professional association; Fr: French-speaking; Nl: Dutch–speaking, F: female, M: male, ONE: Office national de la naissance et de l’enfance (National center for birth and childhood – French Community), K&G (National center for birth and childhood – Flemish Community)

Reference

1. The Centre for Workforce Intelligence: **Horizon scanning. Analysis of key forces and factors.** In *Centre for Workforce Intelligence technical paper series no 0006* UK; 2014.

1. The definitions of sections are those produced by Willis et al (Reference: The Centre for Workforce Intelligence: **Horizon scanning. Analysis of key forces and factors.** In *Centre for Workforce Intelligence technical paper series no 0006* UK; 2014.) [↑](#footnote-ref-1)
2. The definition of this section is adapted to the Belgian concept [↑](#footnote-ref-2)
